# Trends in the Social Class Inequalities in Disability and Self-Rated Health: Repeated Cross-Sectional Surveys from Finland and Sweden 2001–2018

**DOI:** 10.3389/ijph.2021.645513

**Published:** 2021-05-10

**Authors:** Linda Enroth, Stefan Fors

**Affiliations:** ^1^ Faculty of Social Sciences (Health Sciences) and Gerontology Research Center, Tampere University, Tampere, Finland; ^2^ Aging Research Center, Karolinska Institutet and Stockholm University, Stockholm, Sweden; ^3^ Center for Epidemiology and Community Medicine, Region Stockholm, Sweden

**Keywords:** socioeconomic status, ADL, oldest old, time series, inequality

## Abstract

**Objectives:** To assess time trends in the social class inequalities and in total inequality in disability and self-rated health (SRH) in two oldest old populations.

**Methods:** The data came from the Finnish Vitality 90+ Study (2001, 2003, 2007, 2010, 2014 and 2018; n = 5,440) and from the Swedish Panel Study of Living Conditions of the Oldest Old (2002, 2004, 2011 and 2014; n = 1,645). Inequalities in mobility and activities of daily living (ADL) disability and SRH were examined cross-sectionally and over time using relative and absolute measures.

**Results:** Lower social classes had greater mobility and ADL disability and worse SRH than higher social classes and the inequalities tended to increase over time. Findings were remarkably similar in both studies and with absolute and relative measures. Total inequality, referring to the variance in health outcome in the total population, remained stable or decreased.

**Conclusion:** The study suggests that the earlier findings of improved mobility and ADL are largely driven by the positive development in higher social classes while findings of decline in SRH are related to the worsening of SRH in lower social classes

## Introduction

The largely accepted policy goal to reduce socioeconomic health inequalities has got less attention when it comes to the fast-growing oldest old populations [1]. Health and functioning in old age are characterized by increasing heterogeneity. On one hand, genetic predisposition to health deterioration and on the other, accumulation of social inequality over the life course contributes to greater variability in diseases, disability and mortality in old age [2, 3]. Improved living and working conditions and the educational expansion have greatly contributed to population aging, and together with advances in medical technology increased survival from diseases and disabilities postponing mortality to older ages [4]. The rapid increase in longevity during the past decades may have implications for health and the socioeconomic distribution among older people [5].

Activities of daily living (ADL) and mobility are commonly used indicators of selfcare and a prerequisite for independent living. Self-rated health (SRH) is a more subjective indicator of a general health status [6]. ADL, mobility and SRH are all associated with quality of life, care needs and mortality, and are considered important health indicators in old age [6, 7]. The recent trends for the oldest old populations suggest some improvement in mobility and ADL [8, 9] but worsening in SRH [8, 10] in Finland and Sweden.

People in low socioeconomic groups tend to have more ADL and mobility limitations, more diseases and worse SRH than people in higher socioeconomic groups [8, 11, 12]. As survival with such morbidities has increased and as they are more common in lower socioeconomic groups, this development could lead to the appearance of increasing health inequalities. Concurrently, educational expansion leading to decreased social selection to higher socioeconomic positions, may have changed the composition of socioeconomic groups leaving the group with least education smaller but more negatively selected [13].

Mackenbach and Kunst (1997) [14] as well as others [15, 16], have highlighted that examination of time trends in the magnitude of socioeconomic inequalities in health should include both absolute and relative measures. First, because absolute measures provide means to estimate the magnitude of the health problem (i.e. the burden at population level) while relative measures pinpoint the existence of inequality regardless of the absolute magnitude [17]. Second, they may exhibit diverging trends. In case of either simultaneous increase or decrease in the prevalence of a health problem in compared groups, the trends for absolute and relative inequality could move in opposite directions [16].

Research on time trends in socioeconomic health inequalities has systematically found either increasing or constant inequalities in ADL, mobility and SRH during the period 1990–2014. This applies to educational [8, 18–23], social class [23], and income [18, 22, 24, 25] inequalities in health among people aged 65+ in Europe, US and Asia. In addition to predefined population groups (e.g. between socioeconomic groups), heterogeneity in health has recently been discussed and analyzed in terms of total inequality in health, referring to the variance of health across all individuals [3, 26, 27].

A considerable number of studies have examined health inequalities in old age, and some have studied trends over time. However, very few have focused on the oldest old population or utilized several health indicators over more than three time points. This study examines trends in the social class differences in disability and self-rated health and in total inequality in the oldest old populations of Finland and Sweden between 2001 and 2018. We use data from two independent samples: Vitality 90+ Study and SWEOLD, and analyze trends in ADL, mobility and SRH by occupational social class using both absolute and relative measures. By using data from two neighboring Nordic countries, we are able to assess the robustness and generalizability of the findings across the region.

## Methods

### Data

The Vitality 90+ Study and the Swedish Panel Study of Living Conditions of the Oldest Old (SWEOLD) are large population-based survey studies with several cross-sectional data collections over the last two decades. The Vitality 90+ Study encompasses everyone aged 90 years and over residing in the third largest city of Finland (Tampere) in 2001, 2003, 2007, 2010, 2014 and 2018. The data were collected using a mailed questionnaire and have response rates between 77-86% over the years [9]. There were in total 5,440 participants with 7,589 observations between 2001 and 2018 of which 78% were women. For the analyses, we included 6,406 observations with information on social class. The SWEOLD survey were based on random samples of the Swedish population aged 77 and over. The interviews were conducted face-to-face (in 2002 and 2011) or via telephone (in 2004 and 2014) [28]. Response rates varies between 84–87%. There were in total 1,645 participants with 2,893 observations between 2002 and 2004 of which 58% were women. For the analyses, we included 2,625 observations with information on social class. Both studies include individuals living at home and in round-the-clock care and they allow the use of proxy respondents. The study protocols were approved by the Regional Ethics Committee of Tampere University Hospital or The Regional Ethics Review Board in Stockholm.

### Independent Variable

#### Occupational Social Class

The participants’ longest held own occupation was used as the measure of a social class. Social class has been shown to be an applicable measure of social stratification also in older populations and produce equivalent findings for women either with own or spouse’s occupation [29–31]. Individual occupations were categorized into four hierarchical classes according to their social and economic characteristics [32]. For the Finnish data, the classification was based on the International Standard Classification of Occupations [33]. For the Swedish data, the classification was based on the Swedish Socioeconomic Classification System (SEI) developed by Statistics Sweden [34]. The four social classes were upper non-manual workers, lower non-manual workers, skilled manual workers and unskilled manual workers. In addition, a rank based linear predictor was formed of the social class categories with scaling from zero (highest level) to one (lowest level) [35–37].

### Outcome Variables

#### Disability and Self-Rated Health

Mobility was assessed by the self-reported ability to climb stairs and walk 100/400 m, and additionally by the ability to move indoors in Vitality 90+ Study. Activities of daily living (ADL) were assessed by the self-reported ability to get in and out of bed and to dress and undress. In the Vitality 90+ Study, the response options were: 1) Yes, without difficulty, 2) Yes, with difficulty, 3) Only with help, or 4) Not at all. Respondents were considered disabled in mobility and ADL if they chose response options 3 or 4 for at least one mobility or ADL activity respectively. In SWEOLD, the response options for mobility were: 1) Yes, or 2) No, and for ADL: 1) Yes, manage completely by myself, 2) Yes, with help, or 3) No, not at all. Respondents who did not manage both activities without help of another person (response option 2 in mobility and 2 and 3 in ADL) were considered disabled in mobility and ADL. Self-rated health (SRH) was assessed with the question: “How would you evaluate your present health status?” Of the five alternative answers in Vitality 90+ Study: 1) very good, 2) fairly good, 3) average, 4) fairly poor and 5) poor, the two last alternatives were classified as poor SRH. Of the three alternative answers in SWEOLD: 1) good, 2) neither good nor poor and 3) poor, the last alternative was classified as poor SRH. Proxy interviews were excluded from the SRH analyses due to the subjective nature of the question. Furthermore, to examine the total inequality in disability and SRH, all three outcomes were treated as continuous variables. In the Vitality 90+ Study, the range was 2–8 in ADL, 3–12 in mobility and 1-5 in SRH. In SWEOLD, the corresponding ranges were 2–6 in ADL, 2–4 in mobility and 1–3 in SRH.

### Statistical Analysis

First, we examined the absolute inequalities in ADL and mobility disability and poor SRH by social class in each study year with unadjusted and age and sex adjusted predicted probabilities. Second, we examined the population attributable risk (PAR) in each study year using a regression-based measure of PAR (*regpar with subpop command* in Stata) where upper non-manual workers were used as the reference category [38]. PAR shows a theoretical percent of people who would be prevented from disability or poor SRH if all subjects had the risk of the highest social class. Third, we examined the relative inequalities in the three outcomes with a regression-based summary measure of relative index of inequality (RII). Age and sex adjusted RII was analyzed using generalized linear models with log-binomial regression models. RII is a rate ratio of the outcome between the theoretical bottom and top of the social class hierarchy [36].

To examine time trends in inequalities in ADL and mobility disability and poor SRH, we analyzed interaction terms between social class and study year using RII. Furthermore, we examined sex differences in inequalities by interaction terms between social class and sex. To control for multiple participation, a cluster-correlated robust estimate of variance was used in the analyses with interaction terms.

In addition to social class inequalities, we examined total inequality. We tested the equality of standard deviations for disability and poor SRH in the total population to examine whether the variance changed over time. We ran Levene’s robust test statistic for skewed outcomes and used Brown and Forsythe (1974) [39] proposed medians for interpretation of statistical significance.

For the SWEOLD data, weights were used to correct for oversampling of 85 + population (*pweight* in Stata) in all analyses except the analyses of total inequalities. The analyses were conducted with Stata 16.

## Results

### Vitality 90+ Study (V)

The analytical sample consisted of 1,565 men and 4,841 women with mean age of 92.5 years. The social class structure included upper non-manual workers (11.9%), lower non-manual workers (36.0%), skilled manual workers (40.6%) and unskilled manual workers (11.5%). The proportion of proxy answers was 16.9% and 33.2% of the participants resided in long-term care facility. From 2001 to 2018, there was an increase in mean age (92.2 vs. 92.7), proportion of men (23.0 vs. 27.7%) and non-manual workers (38.8 vs. 52.4%) but a decrease in the proportion of long-term care residents (35.8 vs. 29.0%) (Table 1).

**TABLE 1:** Description of the study samples in the Vitality 90+ Study (2001–2018) and Swedish Panel Study of Living Conditions of the Oldest Old (SWEOLD) (2002–2014).

	The Vitality 90+ Study (Finland)						SWEOLD (Sweden)			
Study year	2001	2003	2007	2010	2014	2018	2002	2004	2011	2014
n	653	730	752	1,102	1,444	1,725	549	540	839 (Weighted 593)	697 (Weighted 628)
Response rate of the survey	79.0	86.3	82.3	79.5	79.6	76.7	84.4	87.3	86.2	84.3
Age										
** **Mean	92.2	92.3	92.5	92.5	92.6	92.7	83.2	83.2	86.4	84.5
** **Min, Max	90,102	90,106	90,105	90,107	90,106	90,107	77,99	77,100	77,101	77,105
Sex, %										
** **Women	77.0	79.0	79.0	79.0	74.7	72.3	54.3	55.2	61.0	58.4
** **Men	23.0	21.0	21.0	21.0	25.3	27.7	45.7	44.8	39.0	41.6
Social class, %										
** **Upper non-manual	12.3	8.2	10.0	8.2	13.6	14.1	20.2	20.0	24.7	30.2
** **Lower non-manual	26.5	23.6	35.0	39.5	38.0	38.3	24.2	24.4	25.9	23.4
** **Skilled manual	49.6	36.1	44.7	43.4	36.4	34.1	25.9	23.9	21.0	20.3
** **Unskilled manual	11.6	8.0	10.4	9.0	12.0	13.5	29.7	31.7	28.4	26.1
Respondent, %										
** **Participant/mixed	81.4	82.0	86.8	79.7	82.4	85.3	81.8	81.7	81.3	83.7
** **Proxy	18.6	18.0	13.2	20.3	17.6	14.7	18.2	18.3	18.7	16.3
Place of stay/residency, %										
** **Home	64.2	66.85	66.13	63.9	65.7	71.0	86.5	89.3	89.6	87.6
** **Long-term care	35.8	33.15	33.87	36.1	34.3	29.0	13.5	10.7	10.4	12.4

For SWEOLD, percentages are from the weighted numbers.

### SWEOLD (S)

The analytical sample consisted of 1,188 men and 1,437 women with mean age of 84.6 years. The social class structure included upper non-manual workers (24.1%), lower non-manual workers (24.5%), skilled manual workers (22.6%) and unskilled manual workers (28.9%). The proportion of proxy answers was 17.9% and 11.8% of the participants resided in long-term care facility. Mean age (83.2–86.4), proportion of men (39.0–45.7%), proxy respondents (16.3–18.7%) and long-term care residents (10.4–13.5%) slightly varied over time while the proportion of non-manual workers increased from 44.4 to 53.6% (Table 1).

### Absolute Inequalities

**FIGURE 1:**
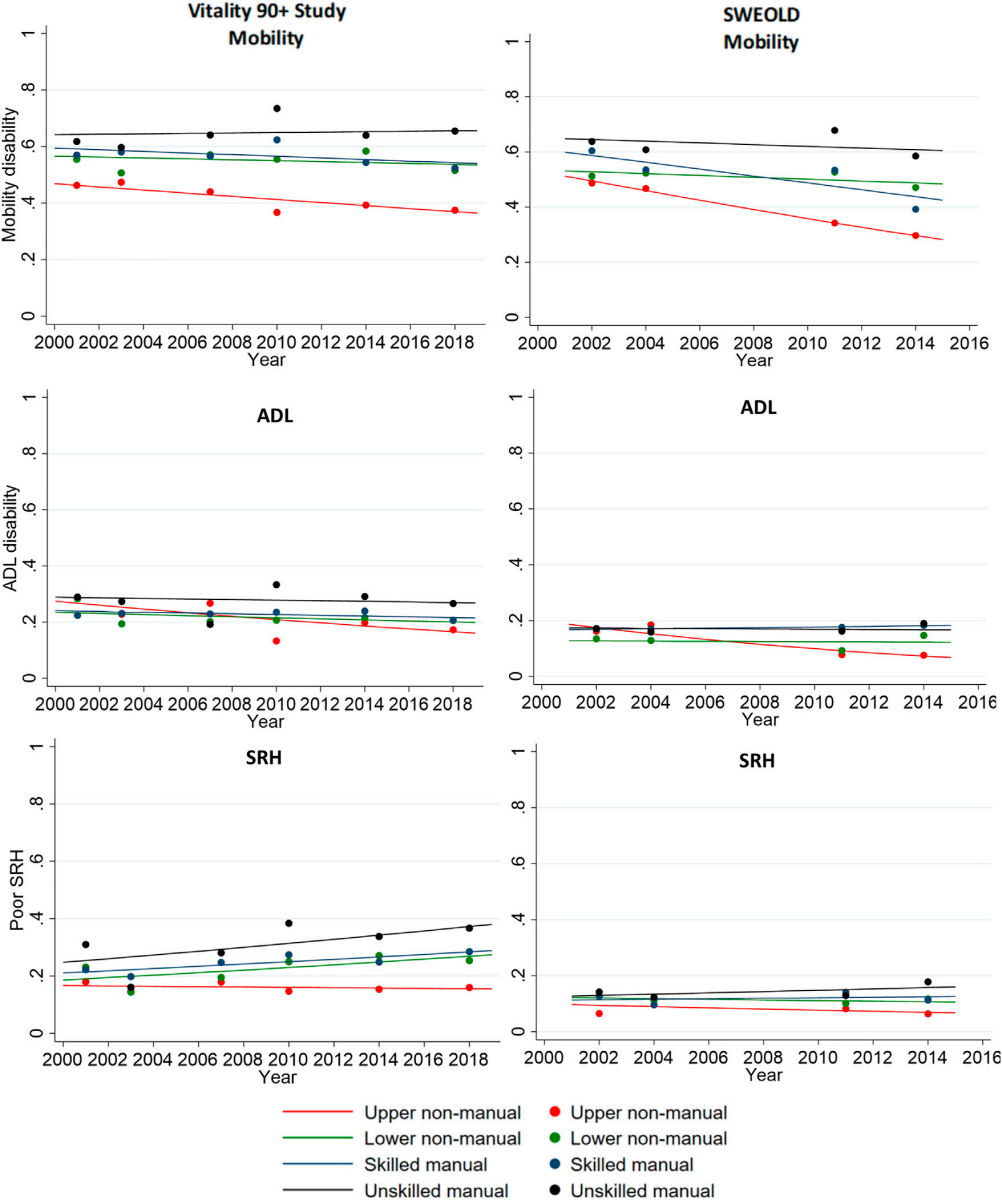
Unadjusted (dot) and age and sex adjusted (line) predicted probabilities of mobility and activities of daily living (ADL) disability and poor self-rated health (SRH) by social class in Vitality 90+ Study (2001–2018, Finland) and Swedish Panel Study of Living Conditions of the Oldest Old (SWEOLD) (2002–2014, Sweden).

The probability of mobility disability was systematically lowest among upper non-manuals and highest among unskilled manuals between 2001 and 2018 in Vitality 90+ Study and between 2002 and 2014 in SWEOLD. In Vitality 90+ Study (V), the social class inequalities were statistically significant in mobility as of 2010, and in SWEOLD (S) in all but 2004 measurement point. Disability in mobility decreased for upper non-manuals (V 46.3 vs. 37.5% and S 48.7 vs. 29.7%) over time but less or not at all for other social classes. There were no inequalities in ADL between 2001-2007, but as of 2010 a social gradient emerged showing lowest ADL disability for upper non-manuals and highest for unskilled manuals. Inequalities were statistically significant in 2010 in Vitality 90+ Study and in the two last study years in SWEOLD. ADL disability decreased over time for all social classes with the greatest decline among upper non-manuals (V 28.8 vs. 17.3% and S 16.2 vs. 7.6%). The probability of poor SRH was lowest for upper non-manuals in all study years. Social class inequalities in SRH were statistically significant as of 2010 in Vitality 90+ Study and in 2014 in SWEOLD. In the Vitality 90+ Study, all social classes except the upper non-manuals exhibited an increase in poor SRH over the study period. In SWEOLD, the probability of poor SRH had some variation across social classes and showed a slight increase over time among unskilled manuals (Figure 1; Supplementary Table S1).

### Population Attributable Risk

**FIGURE 2:**
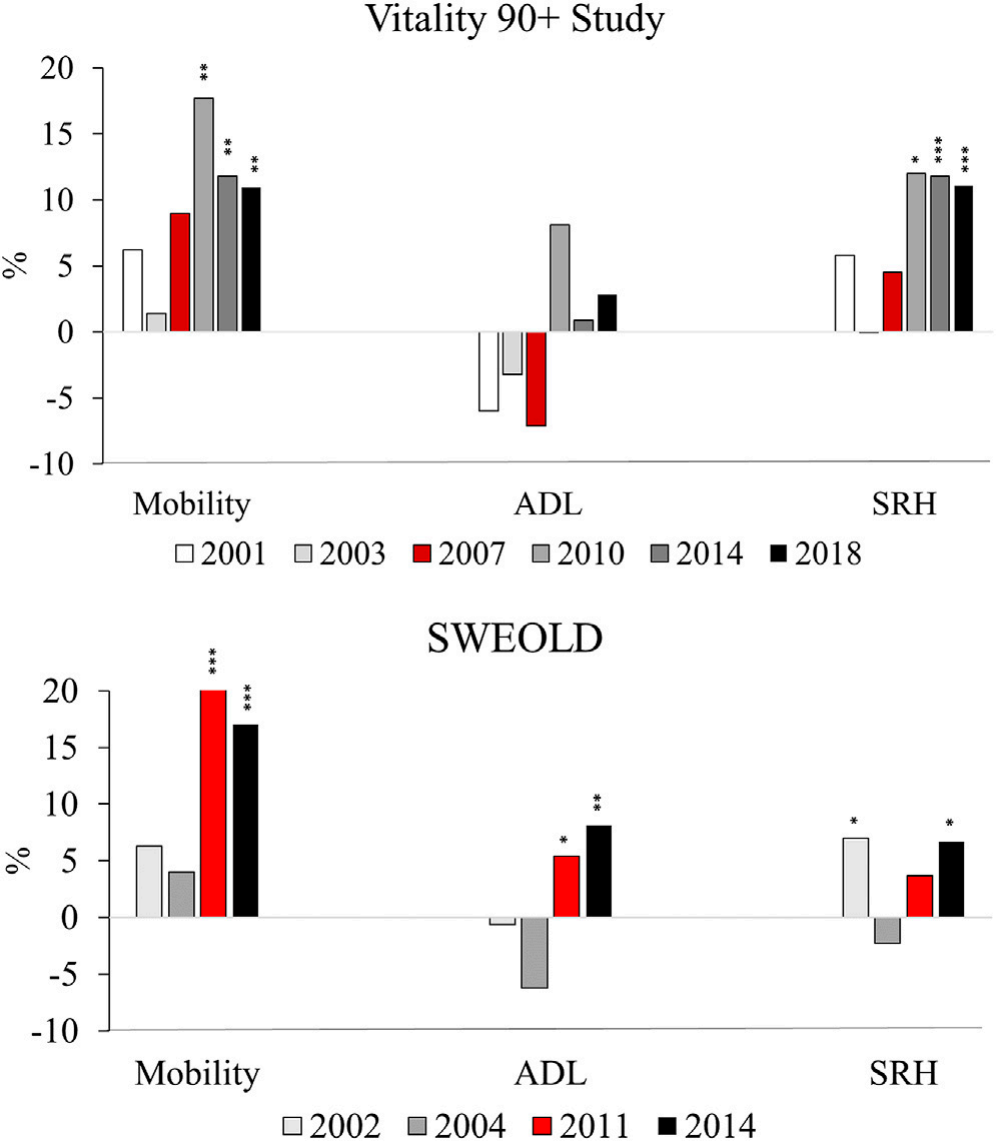
Population attributable risk (PAR) of mobility and activities of daily living (ADL) disability and poor self-rated health (SRH) in Vitality 90+ Study (2001–2018, Finland) and SWEOLD (2002–2014, Sweden). *p*-values *< 0.05, **< 0.01, ***< 0.001.

If all social classes had the health status of upper non-manuals, the overall burden of mobility disability and poor SRH would have been decreased by 0–9% between 2001-2007 and by more than 10% between 2010–2018 according to the Vitality 90+ Study. The estimated PAR was statistically significant for mobility and SRH as of 2010. For ADL, upper non-manuals had higher probability of disability than the other social classes between 2001–2007 and lower between 2010–2018. However, the estimated PAR was not statistically significant for ADL. In SWEOLD, the PAR was around 5% in mobility in the beginning of the study period and higher than 15% during the last two study years showing statistically significant differences. Similarly, the estimated PAR was statistically significant for ADL (5%) in 2011 and (8%) 2014. The burden of poor SRH would have been 7% lower (2002 and 2014) if all classes had the SRH prevalence of the upper non-manuals. (Figure 2; Supplementary Table S2).

### Relative Inequalities

**FIGURE 3:**
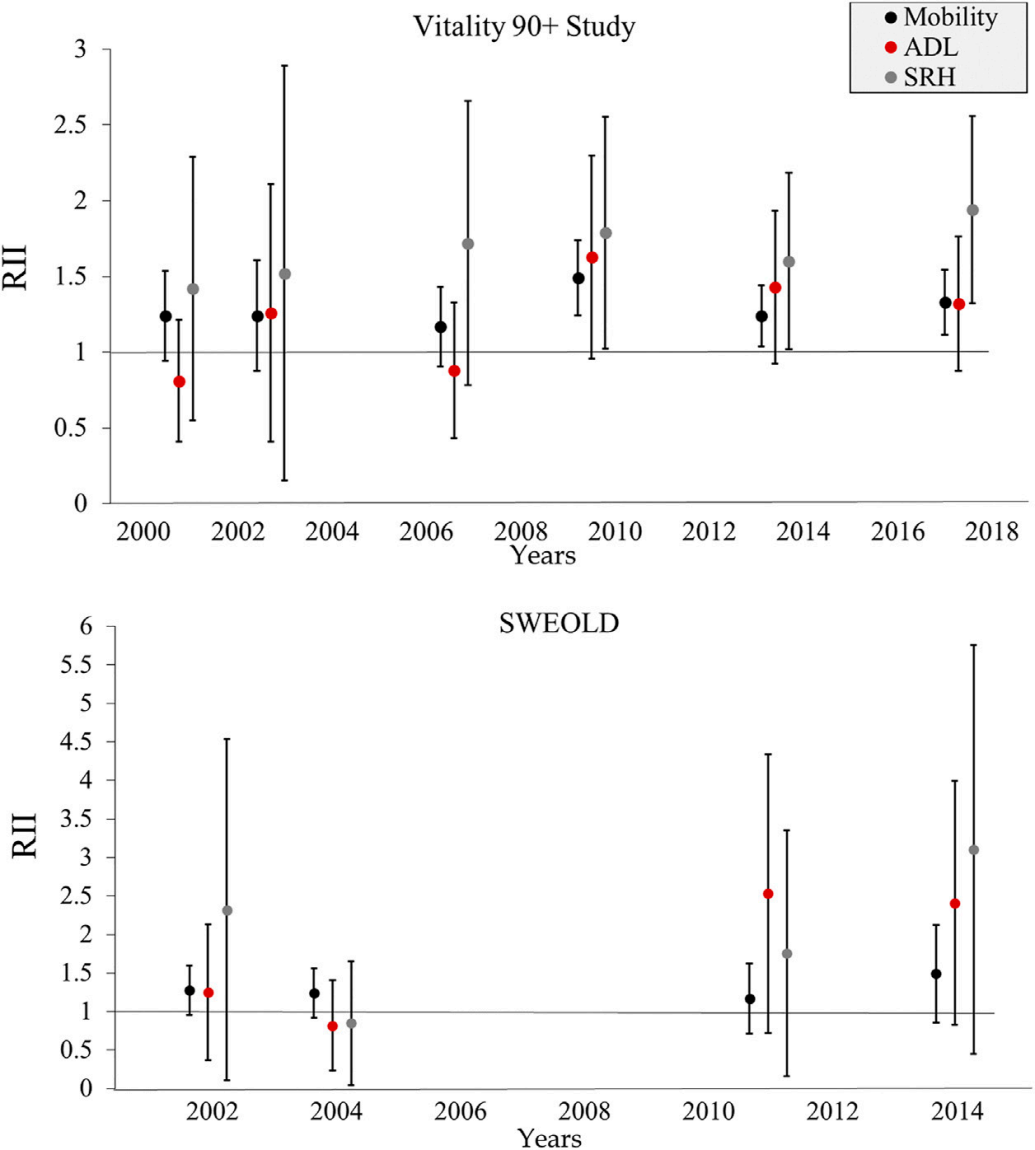
Age and sex adjusted relative index of inequality (RII) for mobility and activities of daily living (ADL) disability and poor self-rated health (SRH) in Vitality 90+ Study (2001–2018, Finland) and SWEOLD (2002–2014, Sweden). RII values higher than 1 denote higher prevalence of disability and poor SRH among lower compared with higher social classes. Note the different scales.

In Vitality 90+ Study, the age and sex adjusted relative index of inequality (RII) was higher than one for mobility and SRH at each point of measurement, indicating a health advantage for the higher social classes. The RII estimate was statistically significant for mobility as of 2010, for SRH as of 2007, and for ADL in 2010 and 2014. In SWEOLD, the RII was higher than one for each study year and each health outcome except for ADL and SRH in 2004. The RII estimate was statistically significant for mobility and ADL in 2011 and 2014 and for SRH in 2014 (Table 2; Figure 3).

**TABLE 2:** Age and sex adjusted relative index of inequality (RII) for mobility and activities of daily living (ADL) disability and poor self-rated health (SRH) in Vitality 90+ Study (2001–2018, Finland) and SWEOLD (2002–2014, Sweden). RII for interaction terms between social class and study year, and for social class and sex. RII values higher than 1 denote higher prevalence of disability and poor SRH among lower compared with higher social classes. CI = confidence interval.

Study year	The Vitality 90+ Study (Finland)						SWEOLD (Sweden)			
	2001	2003	2007	2010	2014	2018	2002	2004	2011	2014
	RII Cis	RII CIs	RII CIs	RII CIs	RII CIs	RII CIs	RII CIs	RII CIs	RII CIs	RII CIs
Mobility^Model1^	1.24	1.24	1.17	1.49***	1.24*	1.33***	1.28	1.16	1.71***	2.09***
	0.98–1.57	0.92–1.66	0.93–1.46	1.26–1.76	1.05–1.45	1.14–1.56	0.99–1.64	0.88–1.52	1.31–2.23	1.55–2.83
Social class × study year^Model2^	1.00						1.04**			
	0.99–1.02						1.01–1.07			
Social class × study year^Model3^	Reference	1.05	0.90	1.24	0.99	1.11	Reference	1.00	1.43*	1.58*
		0.74–1.51	0.64–1.26	0.91–1.68	0.74–1.34	0.82–1.49		0.74–1.35	1.02–2.00	1.07–2.34
Social class × sex^Model4^	1.08	0.97	0.59	0.78	1.04	0.68	0.92	0.96	0.71	1.29
	0.49–2.39	0.35–2.67	0.28–1.23	0.42–1.45	0.64–1.69	0.43–1.08	0.52–1.62	0.52–1.80	0.42–1.19	0.66–2.49
ADL^Model1^	0.81	1.26	0.88	1.63*	1.43*	1.32	1.25	0.82	2.53*	2.41**
	0.49–1.33	0.64–2.47	0.53–1.47	1.08–2.45	1.00–2.03	0.94–1.84	0.62–2.53	0.40–1.68	1.24–5.16	1.25–4.64
Social class × study year^Model2^	1.02						1.09*			
	0.99–1.05						1.01–1.18			
Social class × study year^Model3^	Reference	1.58	1.09	2.07*	1.73	1.55	Reference	0.79	2.10	2.19
		0.73–3.43	0.52–2.25	1.06–4.04	0.92–3.26	0.83–2.89		0.37–1.71	0.81–5.46	0.86–5.54
Social class × sex^Model4^	2.81	1.67	0.32	0.62	2.07	0.95	1.10	1.95	0.49	0.20*
	0.74–10.72	0.27–10.29	0.08–1.32	0.18–2.11	0.84–5.09	0.43–2.11	0.27–4.51	0.43–8.84	0.13–1.88	0.05–0.77
SRH^Model1^	1.42	1.52	1.72*	1.79**	1.60*	1.94***	2.32	0.85	1.76	3.10**
	0.77–2.62	0.62–3.73	1.00–2.96	1.17–2.74	1.11–2.30	1.41–2.66	0.89–6.00	0.33–2.18	0.72–4.35	1.32–7.28
Social class × study year^Model2^	1.01						1.07			
	0.97–1.04						0.97–1.17			
Social class × study year^Model3^	Reference	1.07	1.17	1.24	1.08	1.36	Reference	0.51	0.91	1.81
		0.41–2.84	0.52–2.62	0.58–2.66	0.53–2.21	0.68–2.73		0.15–1.71	0.27–3.03	0.54–6.06
Social class × sex^Model4^	1.10	0.58	0.30	1.33	0.83	0.78	1.40	3.71	0.54	0.88
	0.25–4.80	0.05–6.63	0.07–1.21	0.46–3.81	0.36–1.91	0.35–1.70	0.20–9.66	0.41–33.22	0.10–3.04	0.13–6.21

Model 1: age and sex adjusted relative index of inequality; Model 2: age and sex adjusted relative index of inequality for interaction terms between social class and study year (change over time); Model 3: age and sex adjusted relative index of inequality for interaction terms between social class and study year (social class inequalities in comparison to the first study year); Model 4: age adjusted relative index of inequality for interaction terms between social class and sex in each study year.

*p*-values *<0.05, **< 0.01, ***< 0.001.

### Time Trends and Sex Inequalities

The interaction terms showed no statistically significant trends in the magnitude of social class inequalities in mobility, ADL or SRH between 2001 and 2018 in the Vitality 90+ Study. However, as compared with the measurement point in 2001, the inequalities were larger for ADL in 2010 (RII 2.07; CI 1.06–4.04). Based on the interaction terms between social class and sex, there were no statistically significant differences in social class inequalities between women and men. In SWEOLD, the social class inequalities increased for mobility (RII 1.04; CI 1.01–1.07) and ADL (RII 1.09; CI 1.01–1.18) between 2002 and 2014. Tested with interaction terms, the magnitude of the social class inequalities differed between women and men in one case. In 2014, men had greater inequalities in ADL than women (RII 0.20; CI 0.05–0.77) (Table 2).

### Total Inequality in Disability and SRH

We tested whether the total variance of disability and SRH changed over time in the populations. In Vitality 90+ Study, the standard deviations around the mean decreased for mobility (*p*-value 0.001) and ADL (*p*-value <0.001). There were no statistically significant differences in SWEOLD (Table 3).

**TABLE 3:** Total inequality in mobility and ADL disability and poor self-rated health in the Vitality 90+ Study (2001–2018, Finland) and Swedish Panel Study of Living Conditions of the Oldest Old (SWEOLD) (2002–2014, Sweden). SD = standard deviation.

	The Vitality 90+ Study (Finland)						
Study year	2001	2003	2007	2010	2014	2018	*p*-value
	Mean (SD)	Mean (SD)	Mean (SD)	Mean (SD)	Mean (SD)	Mean (SD)		
Mobility disability	6.99 (3.10)	6.81 (3.08)	6.79 (2.91)	6.89 (2.94)	6.77 (2.99)	6.52 (2.88)	0.001
ADL disability	3.62 (2.11)	3.45 (2.01)	3.46 (1.90)	3.49 (1.92)	3.50 (1.93)	3.27 (1.77)	<0.001
Poor self-rated health	2.89 (0.93)	2.80 (0.85)	2.92 (0.86)	3.03 (0.88)	3.00 (0.89)	3.01 (0.88)	0.32
	**SWEOLD**						
	2002	2004		2011	2014		
Mobility disability	2.91 (0.87)	2.84 (0.85)		2.93 (0.88)	2.74 (0.87)		0.47
ADL disability	2.27 (0.65)	2.27 (0.65)		2.28 (0.66)	2.28 (0.66)		0.98
Poor self-rated health	1.66 (0.68)	1.67 (0.67)		1.61 (0.67)	1.64 (0.67)		0.53

## Discussion

This study examined trends in social class inequalities in disability and self-rated health between 2001 and 2018 in two studies of the oldest old from the Nordic countries, the Vitality 90+ Study and SWEOLD. The main findings show greater relative inequalities in disability and SRH for more recent years in both studies. Lower social classes had more mobility and ADL disabilities as of 2010 and worse SRH as of 2007 in the Vitality 90+ Study and in 2014 in SWEOLD. The absolute inequalities were mainly in line with the relative inequalities. However, the trend analysis based on interaction terms between social class and study year showed increasing inequalities only for mobility and ADL disability in SWEOLD. Mobility disability declined in all social classes over time but as the decline was steepest among the upper non-manuals, the class inequalities increased during the study period. Similarly, ADL disabilities declined but mostly so among upper non-manuals, which led to increasing inequalities. For SRH we observed an increase in the inequalities due to a worsening trend in the lower social classes. We also examined the total inequalities in disability and SRH over time. The findings show decreasing total inequalities in mobility and ADL disability in Vitality 90+ Study and stability across all outcomes in SWEOLD.

### Mobility

This study is among the first to examine trends in inequalities in disability and SRH among the oldest old populations. Thus, it is difficult to compare our findings with other studies as they are mainly concerned with younger age groups. However, our findings are partly in line with a recent European study, which found an increase in the inequalities in functional limitations in Sweden and constant inequalities in Finland among older adults aged 60+ during the period 2002–2014 [25]. We found increasing inequalities in mobility disability in SWEOLD and greater inequalities in the later study years in the Vitality 90+ Study even if the trend was not statistically significant. Two studies from the United Kingdom have also reported increasing inequalities in mobility over time, although among younger old age groups [21, 22].

### Activities of Daily Living

Previous studies from Finland [19] and Sweden [8] observed inequalities in ADL disability but rather than increasing inequalities as in this study, they found stable inequalities over time. There are some differences between the studies as the previous studies used education instead of the social class as the socioeconomic indicator, the Finnish study was based on a younger population (65–84) and the current study uses more recent data. In line with the findings of this study, a study from the US also found increasing inequality in ADL disability over time [18].

### Self-Rated Health

Earlier findings from Finland [20] and Sweden [8] show constant inequality in SRH over the periods 1993–2003 and 1992–2011, respectively. Similarly, this study did not find statistically significant changes in the magnitude of the inequalities in SRH over time, but indications of greater inequalities in the later study years. Constant or increasing inequalities in SRH was also reported in a European study including 17 countries [23] and in a study of a South Korean population [24].

It has been suggested that the changes in the socioeconomic distribution and the changes in the selection processes into social classes over time could have effects on the magnitude of health inequalities [13]. During the study period from 2001 to 2018, we found increases in the social class inequalities for all three outcomes, but also changes in the social class distributions; as the proportion of non-manual workers increased in both studies. To account for the changes in the socioeconomic distribution, analyses were conducted with a regression-based summary measure of relative index of inequality (RII). RII is recommended for examination of the magnitude of socioeconomic inequality in health over time and for cross-country comparisons [32, 35–37, 40]. Thus, the finding of larger inequalities from 2010 onwards is not likely to be a result of the change in the social class distribution. However, we cannot rule out the possibility of changing selection processes into the social classes. The other mechanisms behind the increasing inequalities could be related to increased survival among the lower social classes in more recent years given that individuals with poorer health would live longer. On the other hand, changes in living conditions such as improved accessibility could lead to decreasing disability, but also to the increases in inequality if the changes were disproportionally beneficial to the higher social classes.

The well-known sex differences in function exist also among the oldest old. Men have better function than women, however there is little evidence on whether socioeconomic inequalities in health differs between sexes in very old age [11]. Tested with interaction terms between social class and sex, we found one difference in ADL in SWEOLD (2014). The social class inequalities were in the same direction among men and women, but the inequalities were greater among men. The intersectionality of sex and social class was not in the scope of this study. However, as it is known that both health and social class distributions are gendered [32], more research on the time trends of sex disparities in social class inequalities in health is needed.

In addition to increasing social class inequalities, we found decreasing total inequalities in disability in the Vitality 90+ Study and stability in SWEOLD. The decreasing total inequalities were mainly driven by the decreasing variance in the higher classes. Hidden by the overall stability in the total inequalities, we also found some decrease in higher classes and an increase in lower classes in SWEOLD. In line with our finding, research on lifespan variation has shown higher variation among people with lower education than among higher educated [27]. Greater variation reflects more uncertainty and heterogeneity in the lower classes while the upper classes are becoming increasingly homogeneous in terms of late-life health.

We found evidence of increasing social class inequalities in disability and SRH in two independent oldest old populations. The Vitality 90+ Study encompasses population-based data on 90 years and older in the third largest city of Finland. The demographic characteristics in the city of Tampere; a doubling of the 90+ population; an increase in the relative proportion of 90+ population; and the sex distribution resembles the overall demographic changes in Finland between 2001 and 2018 [9]. The oldest old populations in both Finland and Tampere are homogenous in terms of ethnic background and language [10], and Tampere encompasses both urban and rural areas. Thus, we believe that the Vitality 90+ Study represents the 90+ population in Finland quite well. The SWEOLD data are based on random samples of the 77+ population in Sweden. As Nordic countries, Finland and Sweden share largely similar traditions and norms and are characterized as welfare states with generous, universal health and social care systems. The results are likely to be generalizable to older adults in Finland and Sweden.

The strengths of the study are the use of data from two oldest old populations with high response rates including people living in round-the-clock care and covering the period from 2001 to 2018. In addition, three different health outcomes were analyzed with similar variables across several cross-sections, using both absolute and relative measures. However, the health and disability information were self-reported and might be affected by changes in health expectations and improved health in age peers. Furthermore, the repeated cross-sectional nature of the study does not allow for causal interpretations.

## Conclusion

This study shows that the increasing longevity in Finland and Sweden has been accompanied by increasing social class inequalities in disability and SRH in the oldest old populations. The increasing inequalities could be attributed to most rapid improvement in mobility and ADL disabilities in the highest classes, and worsening in SRH in lower classes. At the individual level, the development of health and function in old age affects the quality of life, and for ageing societies it has remarkable consequences for health and social care needs. Thus, policies targeting health deterioration and functional decline in groups with low socioeconomic conditions may prove to be an efficient way to simultaneously reduce health inequalities and increase overall health expectancy in old age.

## Data Availability

The data analyzed in this study is subject to the following licenses/restrictions: The data are not freely available but the metadata is described for both data sets online and re-usability is supported. The Vitality 90+ data is described in Finnish Social Science Data Archive and SWEOLD in Swedish National Data Service. Requests to access these datasets should be directed to Vitality 90+ Study: Linda Enroth, Linda.Enroth@tuni.fi: SWEOLD: Carin Lennartsson, Carin.Lennartsson@ki.se.
