# Supplementary material for: Trends in the Social Class Inequalities in Disability and Self-Rated Health: Repeated Cross-Sectional Surveys from Finland and Sweden 2001–2018
Source: Int J Public Health. 2021 May 10;66:645513. doi: 10.3389/ijph.2021.645513 (PMC8565263; doi:10.3389/ijph.2021.645513)
Supplement: Supplementary file 1 [file Table2.DOCX]

**Supplementary material**

**STable 1** Unadjusted prevalence of mobility and activities of daily living (ADL) disability, and poor self-rated health (SRH) by social class. The Vitality 90+ Study (2001-2018) and SWEOLD (2002-2014)

|  | **The Vitality 90+ Study** | | | | | |  | **SWEOLD** | | | | | |
| --- | --- | --- | --- | --- | --- | --- | --- | --- | --- | --- | --- | --- | --- |
|  | 2001 | 2003 | 2007 | 2010 | 2014 | 2018 |  | 2002 | 2004 | 2011 | 2011 (weight) | 2014 | 2014 (weight) |
|  | Mobility disability | | | | | |  | Mobility disability | | | | | |
| n | 650 | 724 | 748 | 1,090 | 1,431 | 1,702 |  | 541 | 539 | 828 | 587 | 683 | 618 |
| Social class, % |  |  |  |  |  |  |  |  |  |  |  |  |  |
| Upper non-manual | 46.3 | 47.4 | 44.0 | 36.7 | 39.3 | 37.5 |  | 48.7 | 46.7 | 40.5 | 34.2 | 31.5 | 29.7 |
| Lower non-manual | 55.5 | 50.7 | 57.1 | 55.5 | 58.4 | 51.6 |  | 51.2 | 52.3 | 56.4 | 52.7 | 50.3 | 47.1 |
| Skilled manual | 57.0 | 58.1 | 56.6 | 62.4 | 54.4 | 52.4 |  | 60.4 | 53.5 | 60.4 | 53.4 | 42.0 | 39.2 |
| Unskilled manual | 61.8 | 59.7 | 64.1 | 73.5 | 64.0 | 65.5 |  | 63.8 | 60.8 | 68.4 | 67.8 | 60.9 | 58.5 |
| Chi-squared test | 0.21 | 0.14 | 0.09 | <0.001 | <0.001 | <0.001 |  | 0.035 | 0.13 | <0.001 |  | <0.001 |  |
|  | ADL disability | | | | | |  |  | ADL disability | | | | |
| n | 651 | 729 | 751 | 1,099 | 1,439 | 1,721 |  | 548 | 539 | 824 | 587 | 687 | 622 |
| Social class, % |  |  |  |  |  |  |  |  |  |  |  |  |  |
| Upper non-manual | 28.8 | 22.8 | 26.7 | 13.3 | 19.8 | 17.3 |  | 16.2 | 18.5 | 12.9 | 7.8 | 9.3 | 7.6 |
| Lower non-manual | 28.3 | 19.4 | 20.2 | 20.7 | 21.6 | 20.6 |  | 13.5 | 12.9 | 13.8 | 9.3 | 16.8 | 14.7 |
| Skilled manual | 22.4 | 23.1 | 22.9 | 23.5 | 23.9 | 20.7 |  | 17.0 | 17.0 | 21.1 | 17.6 | 18.9 | 18.3 |
| Unskilled manual | 29.0 | 27.3 | 19.2 | 33.3 | 29.1 | 26.6 |  | 17.2 | 15.9 | 20.6 | 16.2 | 21.2 | 19.0 |
| Chi-squared test | 0.35 | 0.50 | 0.58 | 0.007 | 0.13 | 0.09 |  | 0.83 | 0.66 | 0.044 |  | 0.01 |  |
|  | Poor SRH | | | | | |  | Poor SRH | | | | | |
| n^a^ | 518 | 595 | 644 | 861 | 1,157 | 1, 420 |  | 449 | 441 | 623 | 479 | 570 | 525 |
| Social class, % |  |  |  |  |  |  |  |  |  |  |  |  |  |
| Upper non-manual | 18.0 | 14.7 | 17.9 | 14.7 | 15.4 | 16.0 |  | 6.5 | 12.0 | 8.5 | 8.2 | 6.0 | 6.4 |
| Lower non-manual | 23.1 | 14.4 | 19.5 | 25.0 | 27.1 | 25.4 |  | 12.5 | 11.2 | 9.5 | 10.1 | 11.6 | 11.7 |
| Skilled manual | 22.2 | 19.8 | 24.7 | 27.4 | 24.9 | 28.5 |  | 12.7 | 9.6 | 12.6 | 14.1 | 11.8 | 11.3 |
| Unskilled manual | 31.0 | 16.1 | 28.1 | 38.4 | 33.8 | 36.7 |  | 14.2 | 12.3 | 12.3 | 12.9 | 16.7 | 17.8 |
| Chi-squared test | 0.38 | 0.43 | 0.26 | 0.01 | 0.002 | <0.001 |  | 0.33 | 0.92 | 0.56 |  | 0.025 |  |

^a^Only self-reports are included in self-rated health analysis.

**STable 2** Age and sex adjusted population attributable risk (PAR) of mobility and activities of daily living (ADL) disability, and poor self-rated health (SRH) by social class. The Vitality 90+ Study (2001-2018) and SWEOLD (2002-2014)

|  | **The Vitality 90+ Study** | | | | | |  | **SWEOLD** | | | |
| --- | --- | --- | --- | --- | --- | --- | --- | --- | --- | --- | --- |
|  | 2001 | 2003 | 2007 | 2010 | 2014 | 2018 |  | 2002 | 2004 | 2011 | 2014 |
|  | Mobility disability | | | | | |  | Mobility disability | | | |
| n | 650 | 724 | 748 | 1,090 | 1,431 | 1,702 |  | 541 | 539 | 828 | 683 |
| PAR  CI | 0.06  -0.06; 0.18 | 0.01  -0.10; 0.13 | 0.09  -0.03; 0.20 | 0.18**  0.07; 0.29 | 0.12**  0.04; 0.20 | 0.11**  0.04; 0.18 |  | 0.06  -0.04; 0.17 | 0.04  -0.06; 0.14 | 0.20***  0.11; 0.29 | 0.17***  0.09; 0.25 |
|  | ADL disability | | | | | |  |  | ADL disability | | |
| n | 651 | 729 | 751 | 1,099 | 1,439 | 1,721 |  | 548 | 539 | 824 | 687 |
| PAR  CI | -0.06  -0.17; 0.05 | -0.03  -0.14; 0.08 | -0.07  -0.18; 0.04 | 0.08  -0.00; 0.17 | 0.01  -0.06; 0.08 | 0.03  -0.03; 0.08 |  | -0.01  -0.08; 0.07 | -0.06  -0.15; 0.02 | 0.05*  0.00; 0.11 | 0.08**  0.03; 0.14 |
|  | Poor SRH | | | | | |  | Poor SRH | | | |
| n^a^ | 518 | 595 | 644 | 861 | 1157 | 1420 |  | 449 | 441 | 623 | 570 |
| PAR  CI | 0.06  -0.05; 0.16 | -0.00  -0.11; 0.11 | 0.05  -0.06; 0.15 | 0.12*  0.03; 0.21 | 0.12***  0.05; 0.18 | 0.11***  0.05; 0.17 |  | 0.07*  0.01; 0.13 | -0.02  -0.10; 0.06 | 0.04  -0.02; 0.10 | 0.07*  0.01; 0.12 |

^a^Only self-reports are included in self-rated health analysis

P-value* <0.05, **<0.01, ***<0.001

CI=confidence interval
